# Use of the combination of the preoperative platelet-to-lymphocyte ratio and tumor characteristics to predict peritoneal metastasis in patients with gastric cancer

**DOI:** 10.1371/journal.pone.0175074

**Published:** 2017-04-06

**Authors:** Xiao-dong Chen, Chen-chen Mao, Rui-sen Wu, Wei-teng Zhang, Ji Lin, Xiang-wei Sun, Chu-huai Chi, Neng Lou, Peng-fei Wang, Xian Shen, Guan-bao Zhu, Shu-rong Shen

**Affiliations:** 1 Department of Gastrointestinal Surgery, The First Affiliated Hospital, Wenzhou Medical University, Wenzhou, Zhejiang, China; 2 Department of Gastrointestinal Surgery, The Second Affiliated Hospital, Wenzhou Medical University, Wenzhou, Zhejiang, China; 3 Department of Breast Surgery, Wenzhou integrated traditional Chinese and Western Medicine Hospital, Wenzhou, Zhejiang, China; Istituto di Ricovero e Cura a Carattere Scientifico Centro di Riferimento Oncologico della Basilicata, ITALY

## Abstract

The aims of the present study were to evaluate the predictive value of the platelet-to-lymphocyte ratio for peritoneal metastasis in patients with gastric cancer and to construct an available preoperative prediction system for peritoneal metastasis. A total of 1080 patients with gastric cancer were enrolled in our study. The preoperative platelet-to-lymphocyte ratio and other serum markers and objective clinical tumor characteristics were evaluated by receiver operating characteristic curves. A logistic analysis was performed to determine the independent predictive indicators of peritoneal metastasis. A prediction system that included the independent predictive indicators was constructed and evaluated by receiver operating characteristic curves. Based on the receiver operating characteristic curves, the ideal platelet-to-lymphocyte ratio cutoff value to predict peritoneal metastasis was 131.00. The logistic analysis showed that the platelet-to-lymphocyte ratio was an independent indicator to predict peritoneal metastasis. The area under the receiver operating characteristic curve was 0.599. When integrating all independent indicators (i.e., platelet-to-lymphocyte ratio, invasion depth, lymphatic invasion, pathological type), the prediction system more reliably predicted peritoneal metastasis with a higher area under the receiver operating characteristic curve (0.769). The preoperative platelet-to-lymphocyte ratio was an indicator that could be used to predict peritoneal metastasis. Our prediction system could be a reliable instrument to discriminate between patients with gastric cancer with and those without peritoneal metastasis.

## Introduction

Gastric cancer (GC) is one of the most common malignant tumors and is the main cause of cancer-related mortality globally, particularly in Asian countries[1]. Owing to the lack of symptomatology and specific diagnosis, the 5-year survival rate of the disease is unfortunately extremely low because patients generally have high-stage disease when diagnosed[2]. Among factors leading to a poor prognosis, peritoneal metastasis is an indispensable indicator[3,4]. A previous study[4] demonstrated that peritoneal metastasis, which accounted for 50% of deaths, is the most important contributing factor of mortality in patients with GC.

Gastrectomy and regional lymphadenectomy are the only curative treatment for GC, but these treatments are insufficient for GC with peritoneal metastasis. Furthermore, a preoperative diagnosis of peritoneal metastasis is necessary for the proper selection of neoadjuvant therapy[5] and conversion therapy[6] to avoid unnecessary surgeries that would cause great physical and psychological harm to patients. Therefore, it is of great importance to accurately predict peritoneal metastasis because this factor contributes to the selection of treatment.

Computed tomography (CT), magnetic resonance imaging, and positron-emission tomography integrated with computed tomography (PET-CT) are widely used to predict peritoneal metastasis; however, all have limited success because of their low sensitivity and specificity[7]. Other research suggests that L-dopa decarboxylase can effectively assist in detecting peritoneal metastasis, but its application is widely limited because of its high cost and technical requirements[8]. Serum tumor markers such as carcinoembryonic antigen (CEA) and carbohydrate antigen (CA)-199 are supplementary tools for detecting peritoneal metastasis in GC[9,10]. However, because of their poor specificity and sensitivity, the expression of these markers alone is insufficient for making a diagnosis.

In addition, the use of systemic inflammatory response (SIR) markers such as platelets, neutrophils, lymphocytes, and monocytes has also been widely reported.[11] Furthermore, the neutrophil-to-lymphocyte ratio (NLR) and platelet-to-lymphocyte ratio (PLR) have been confirmed as essential prognostic factors during the treatment of different types of cancer[12–14]. Other investigators[15,16] have even proposed that the NLR and PLR could be independent prognostic factors in patients with advanced GC. However, no studies have reported on the relationship between the PLR, monocyte-to-lymphocyte ratio (MLR), and peritoneal metastasis.

Therefore, the aims of this study were to determine the relationship between peritoneal metastasis and preoperative inflammatory markers, and to construct a more useful score system to help improve preoperative diagnostic accuracy by combining the independent related factors. We found that the preoperative platelet-to-lymphocyte ratio was useful for predicting peritoneal metastasis and that our prediction system could be a reliable instrument to discriminate between patients with GC with and without peritoneal metastasis. An individualized multimodality treatment could thus be provided to patients with GC.

## Materials and methods

### Patients

In this retrospective analysis, the data of 1199 patients with GC who underwent GC surgery at the First Affiliated Hospital of Wenzhou Medical University (Wenzhou, China) from January 2009 to May 2013 were reviewed. All the patients have received Preoperative CT scan and were CT negative for peritoneal metastasis. The following information was collected and recorded: patient’s personal information (i.e., age, sex, body mass index, family history), tumor characteristics (i.e., location, size, pathological type, histopathological differentiation, lymphatic invasion), and blood routine index (i.e., neutrophils, lymphocytes, platelets, monocytes, NLR, and PLR). The histopathological types were defined as “well differentiated” (i.e., type 1), “moderately differentiated” (i.e., type 2), “poorly differentiated” (i.e., type 3), or “undifferentiated” (i.e., type 4). The pathological type was divided into the ulcerative group and the nonulcerative group. The diagnoses were confirmed in all patients by histological examination. The exclusion criteria included (1) history of gastric resection (8 patients), (2) liver disease such as cirrhosis (14 patients), (3) history of other malignancies (15 patients), (4) severe bleeding and autoimmune disease (22 patients), (5) preoperative chemoradiotherapy (2 patients), (6) severe inflammation or hematological system diseases (34 patients), and (7) distant metastasis excluding abdominal metastases (6 patients). Finally, 1080 patients were eventually enrolled in this study. This study was approved by the Ethics Committee of the First Affiliated Hospital of Wenzhou Medical University. All patients were aware of the research and signed the informed consent form.

### Diagnosis of peritoneal metastasis

According to the Japanese Gastric Cancer Treatment Guidelines (15th edition), the diagnostic criteria for peritoneal metastases were as follows: metastases limited to the greater omentum, lesser omentum, anterior lobe of the transverse mesocolon, pancreatic capsule, and spleen; and metastasis in the upper abdominal peritoneum (visceral peritoneum above the transverse position and parietal peritoneum above the umbilicus). These patients with peritoneal metastases were diagnosed by intraoperative frozen section and postoperative pathological diagnosis.

### Cutoff point of the preoperative PLR and NLR

Blood specimens, which were obtained within 7 days before surgery, were preserved in tubes containing ethylenediaminetetraacetic acid. A hemocounter (XE2100; Sysmex Co., Kobe, Japan) was used to calculate the differential leukocyte, neutrophil, platelet, and monocyte counts. We plotted the receiver operating characteristic (ROC) curves. The values with the maximal Youden index were chosen as the cutoff points of the preoperative PLR and NLR. Thus, the patients were divided into two groups, based on the cutoff point.

### Statistical analysis

We performed the Kolmogorov–Smirnov test to determine the normality of continuous parameters such as the neutrophil count, lymphocyte count, platelet count, monocyte count, PLR, NLR, and MLR. The mean and standard deviation values were used for the normal distributed data, whereas the median and interquartile range values were used for the non-normal distributed data. The Mann–Whitney *U* test was used to compare the non-normal distributed variables between the peritoneal metastasis group and the non-peritoneal metastasis group. ROC analysis was conducted to determine the performance of the variables. The relationship between clinicopathologic characteristics and the NLR or PLR were analyzed using the χ^2^-test. In addition, the χ^2^ test was used for univariate analysis of peritoneal metastasis. Based on the univariate analysis results, a multivariate logistic regression analysis was used to calculate the odds ratio (OR) and 95% confidence interval (CI) of the confirmed independent variables. The area under the ROC curve (AUC) was used to compare the scoring system to other clinicopathologic characteristics. A value of *P* < 0.05 was statistically significant. Statistical analyses were performed using SPSS software (version 22.0; SPSS Inc., Chicago, IL, USA).

## Results

### Patient characteristics

As displayed in Table 1, of the 1080 patients enrolled, 839 patients were men and 241 patients were women. The patients’ median age was 64 years and the interquartile range was 57–72 years. Most (72.4%) patients had a tumor larger than 4.75 cm, 704 patients had a tumor in the antrum, and 638 patients had lymphatic invasion. Based on the histopathological results, the tumors in a large proportion of patients were “differentiated” (555 patients), “moderately differentiated” (202 patients), or “undifferentiated” (214 patients). Based on the pathological type, the cancer in most (935) patients was classified as the ulcerative type. Peritoneal metastasis was detected in 101 patients.

**Table 1:** Clinical and Pathological Characteristics.

Variable	
N	1080
Age (years), median (IQR)	64 (57–72)
Sex (male/female)	839/241
BMI (kg/m^2^), median (IQR)	21.48 (19.48–23.66)
Tumor size (cm), median (IQR)	4.0 (2.5–5.0)
Tumor location [n, (%)]	
Antrum	704 (65.1%)
Corpus	173 (16.0%)
Cardia	185 (17.1%)
Whole	13 (1.2%)
Pathological type [n, (%)]	
Ulcerative	939 (86.9%)
Nonulcerative	141 (13.1%)
Histopathological differentiation [n, (%)]	
Highly differentiated	109 (10.1%)
Moderately differentiated	202 (18.7%)
Poorly differentiated	555 (51.4%)
Undifferentiated	214 (19.8%)
Depth of invasion [n, (%)]	
T1/T2	347 (32.1%)
T3/T4	731 (67.7%)
Lymphatic invasion [n, (%)]	
N0	388 (35.9%)
N1	195 (18.1%)
N2	234 (21.7%)
N3	259 (24.0%)
Peritoneal metastasis [n, (%)]	
Yes	101 (9.4%)
No	979 (90.6%)

Data are missing considering the tumor location for 5 patients, pathological type for 4 patients, depth of invasion for 2 patients, and lymphatic invasion for 4 patients. IQR, interquartile range

### The traits of the preoperative inflammatory indicators in patients with GC with peritoneal metastasis

**Fig 1:**
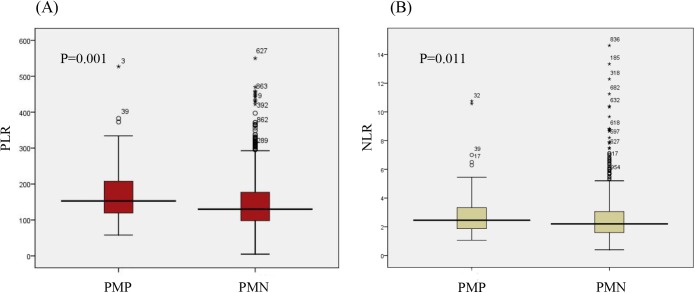
**Distribution of PLR (A) and NLR (B) between PMPG and PMNG**. PLR, platelet-to-lymphocyte ratio; NLR, neutrophil-to-lymphocyte ratio; PMPG, peritoneal metastasis positive group; PMNG, peritoneal metastasis negative group

**Fig 2:**
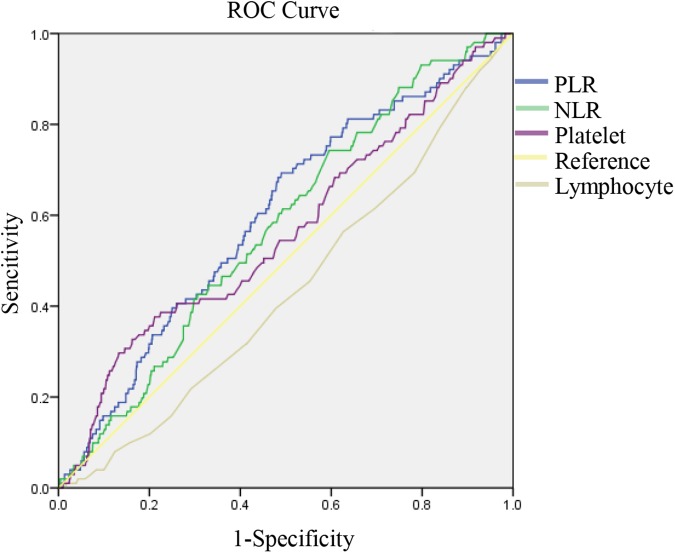
ROC curves for systemic inflammatory response markers in patients with gastric cancer (GC) according to peritoneal metastasis.

As displayed in Table 2, the platelet count was significantly higher in patients with GC with peritoneal metastasis than in patients without peritoneal metastasis (*P* = 0.035). By contrast, the lymphocyte count was higher in patients with GC without peritoneal metastasis (*P* = 0.034). As is shown in Fig 1, the PLR and NLR, which were the combination of two indicators, were also higher in patients with GC with peritoneal metastasis (*P* < 0.05). The ROC curves were then used to further analyze the variables that had a significant difference. As Fig 2 shows, the AUCs of the PLR (0.599, 95% CI 0.543–0.656) and the NLR (0.576, 95% CI 0.522–0.630) were larger than those of the platelet count (0.564, 95% CI 0.502–0.625) and lymphocyte count (0.436, 95% CI 0.379–0.493). This finding indicated that the PLR and NLR were more powerful predictive individual indicators, compared to the other inflammatory indicators.

**Table 2:** Blood Routine Index, According to Peritoneal Metastasis Involvement.

Factors	Total	PMPG	PMNG	*P*
WBC count	6.00 (4.99–7.20)	6.00 (5.05–7.30)	6.00 (4.96–7.16)	0.735
Neutrophil count	3.70 (2.90–4.60)	3.80 (3.15–4.75)	3.70 (2.90–4.60)	0.151
Lymphocyte count	1.60 (1.30–2.00)	1.50 (1.20–1.90)	1.60 (1.30–2.00)	0.034 *
Platelet count	221.00 (179.00–267.00)	227.00 (185.00–307.50)	221.00 (178.00–263.00)	0.035 *
Monocyte count	0.40 (0.30–0.50)	0.40 (0.30–0.50)	0.40 (0.30–0.50)	0.144
NLR	2.24 (1.64–3.07)	2.45 (1.86–3.35)	2.20 (1.60–3.06)	0.011 *
PLR	132.25 (99.14–180.47)	152.78 (118.74–207.56)	130 (98.00–177.27)	0.001 *
MLR	0.285 (0.18–0.35)	0.29 (0.18–0.35)	0.27 (0.16–0.36)	0.642

* Statistically significant (*P* < 0.05).

The values of the variables are presented as the median (IQR). IQR, interquartile range; MLR, monocyte-to-lymphocyte ratio; NLR, neutrophil-to-lymphocyte ratio; PLR, platelet-to-lymphocyte ratio; WBC, white blood cell; PMPG, peritoneal metastasis positive group. PMNG, peritoneal metastasis negative group

### Different clinicopathologic characteristics of GC associated with the NLR and the PLR

The cutoff values of the PLR and NLR for peritoneal metastasis, which were calculated based on the ROC curve, were set to 131.00 and 1.95, respectively (Fig 2). The diagnostic sensitivity and specificity were 69.3% and 51%, respectively, for the PLR and 74.3% and 40.4%, respectively, for the NLR. Based on the cutoff values, patients with GC were divided into the “high PLR group (>131.00)” and the “low PLR group (<131.00)” or the “high NLR group (>1.95)” and the “low NLR group (<1.95).” Among the selected patients, 51.1% of patients had a high PLR and 61.3% of patients had a high NLR. As displayed in Table 3, with regard to the clinicopathologic characteristics examined in our study, a high PLR and NLR were both associated with a larger tumor size, the ulcerative type, deeper invasion, advanced TNM stage, and higher lymphatic invasion status.

**Table 3:** Clinicopathologic Features of Patients, Based on the Platelet-to-Lymphocyte Ratio and Neutrophil-to-lymphocyte Ratio.

Factors	PLR		*P*	NLR		*P*
	>131	<131		>1.95	<1.95	
Sex			0.576			0.27
Men	425	414		529	310	
Women	127	114		133	108	
Age (y)			0.005 *			<0.001 *
>65	380	320		461	239	
<65	172	208		201	179	
Tumor size (cm)			<0.001 *			<0.001 *
>4.75	416	319		480	255	
<4.75	113	168		148	133	
Tumor location			0.298			0.923
Antrum	353	351		436	268	
Corpus	99	74		104	69	
Cardia	94	91		110	75	
Whole	5	8		8	5	
Pathological type			0.586			0.771
Ulcerative	477	462		574	365	
Nonulcerative	75	66		88	53	
Histopathological differentiation			0.754			0.837
Highly differentiated	53	56		64	45	
Moderately differentiated	98	104		120	82	
Poorly differentiated	288	267		345	210	
Undifferentiated	113	101		133	81	
Depth of invasion			<0.001 *			<0.001 *
T1/T2	127	220		181	166	
T3/T4	424	307		479	252	
Lymphatic invasion			<0.001 *			0.009 *
N0	161	227		214	174	
N1	108	87		124	71	
N2	121	113		146	88	
N3	160	99		176	83	
CEA (ng/mL)			0.883			0.87
>5	116	108		139	85	
<5	376	358		451	283	
CA-199 (ng/mL)			0.9			0.389
>35	84	82		107	59	
<35	389	388		473	304	

* Statistically significant (*P* < 0.05).

The values in the table are the number of patients. Data are missing considering the tumor size for 65 patients, tumor location for 5 patients, pathological type for 4 patients, depth of invasion for 2 patients, lymphatic invasion for 4 patients, CEA values for 122 patients, and CA-199 values for 137 patients. CA-199, carbohydrate antigen-199; CEA, carcinoembryonic antigen; NLR, neutrophil-to-lymphocyte ratio; PLR, platelet-to-lymphocyte ratio.

### Univariate and multivariate analysis of the clinicopathologic characteristics

The chi-square test was used to examine the relationship between clinicopathologic characteristics and peritoneal metastasis. As Table 4 shows, tumor location (χ^2^ = 5.142, *P* = 0.0162), tumor size (χ^2^ = 11.481, *P* = 0.001), invasion depth (χ^2^ = 34.635, *P* < 0.001), pathological type (χ^2^ = 4.969, *P* = 0.026), lymphatic invasion (χ^2^ = 63.114, *P* < 0.001), NLR (χ^2^ = 7.89, *P* = 0.005), and PLR (χ^2^ = 14.763, *P* < 0.001) were significantly different between patients with GC with and without peritoneal metastasis. By contrast, no significant differences were detected between the two groups with regard to the body mass index, American Society of Anesthesiologists (ASA) score, sex, CEA level, and CA-199 level. Furthermore, these variables were potential independent risk factors. Logistic regression analyses were performed. As Table 5 shows, the risk of peritoneal metastasis in patients with GC was significantly associated with the PLR (OR = 1.784, *P* = 0.018), invasion depth (OR = 3.630, *P* = 0.009), lymphatic invasion (OR = 7.801, *P* < 0.001), and pathological type (OR = 3.196, *P* = 0.015). Thus, these factors were the independent predictive indicators of peritoneal metastasis.

**Table 4:** Univariate Analysis of the Risk of Peritoneal Metastasis.

Factors	PMPG (n = 101)	PMNG (n = 979)	Univariate analysis	
			χ^2^	*P*
Sex			0.018	0.893
Men	79	760		
Women	22	219		
Age (y)			0.574	0.449
≥65	62	638		
<65	39	341		
BMI			1.934	0.164
≥24	74	759		
<24	27	199		
Albumin (g/L)			3.831	0.05 *
≥35	77	827		
<35	23	151		
ASA score			0.865	0.352
≥3	7	95		
<3	94	870		
Tumor size (cm)			11.481	0.001 *
≥4.75	82	653		
<4.75	12	268		
Tumor location			5.142	0.0162 *
Antrum	64	640		
Corpus	20	153		
Cardia	13	172		
Whole	3	10		
Histopathological differentiation			2.806	0.419
Type 1	8	101		
Type 2	22	180		
Type 3	56	499		
Type 4	15	199		
Pathological type			4.969	0.026 *
Ulcerative type	91	844		
Nonulcerative type	6	135		
Depth of invasion			34.635	<0.001 *
T1/T2	6	341		
T3/T4	94	637		
Lymphatic invasion			63.114	<0.001 *
N0	9	379		
N1	16	179		
N2	22	212		
N3	54	205		
PLR			14.763	<0.001 *
≥131.00	70	482		
<131.00	31	497		
NLR			7.89	0.005 *
≥1.95	75	587		
<1.95	26	392		
CEA (ng/mL)			1.681	0.195
≥5	26	198		
<5	64	670		
CA-199 (ng/mL)			2.608	0.107
≥35	10	156		
<35	78	699		

* Statistically significant (*P* < 0.05).

The values in the table are the number of patients. Data are missing considering the BMI for one patient, ASA score for 8 patients, albumin for 2 patients, tumor size for 65 patients, tumor location for 5 patients, pathological type for 4 patients, depth of invasion for 2 patients, lymphatic invasion for 4 patients, CEA values for 122 patients, and CA-199 values for 137 patients. BMI, body mass index, CA-199, carbohydrate antigen 199; CEA, carcinoembryonic antigen; PLR, platelet-to-lymphocyte ratio; NLR, neutrophil-to-lymphocyte ratio; PMPG, peritoneal metastasis positive group. PMNG, peritoneal metastasis negative group

**Table 5:** Multivariate Analysis to Evaluate Potential Predictive Factors for Peritoneal Metastasis and the Scoring of these Factors.

Factors	Multivariate analysis			Risk score
	OR	95% CI	*P* *	
Pathological type				
Nonulcerative type	1			0
Ulcerative type	3.196	1.255–8.143	0.015	50
Depth of invasion				
T1/T2	1			0
T3/T4	3.630	1.381–9.543	0.009	56
Lymphatic invasion				
N0	1			0
N1	3.752	1.402–10.047	0.008	57
N2	3.733	1.433–9.720	0.007	57
N3	7.801	3.133–19.420	<0.001	89
PLR				
<131	1			0
≥131	1.784	1.105–2.880	0.018	25

* All values in this column are statistically significant (*P* < 0.05).

Data are missing considering the pathological type for 4 patients, depth of invasion for 2 patients, and lymphatic invasion for 4 patients. CI, confidence interval; NLR, neutrophil-to-lymphocyte ratio; PLR, platelet-to-lymphocyte ratio; OR, odds ratio.

### Construction of the scoring system for peritoneal metastasis in GC

**Fig 3:**
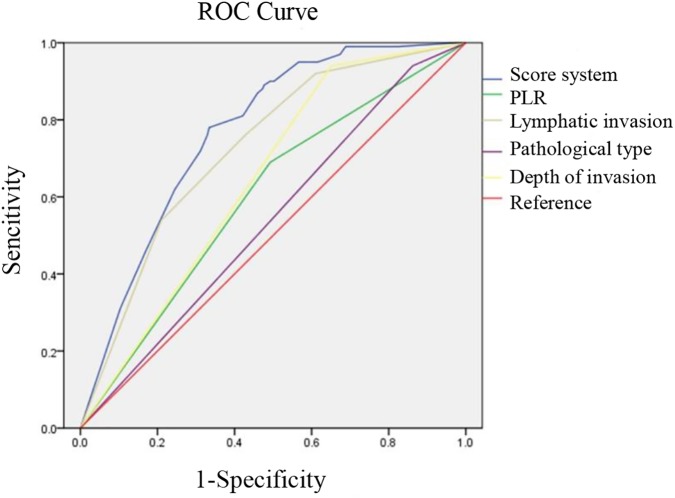
Comparison of ROC curves for depth of invasion, lymphatic invasion, pathological type, PLR, and the score system to evaluate the probability of peritoneal metastasis.

The independent preoperative predictors were selected, according to the multivariate logistic regression analysis. As is shown in Table 5, the risk scores, which were logarithmically transformed from each independent risk factor and multiplied by 100, were calculated and assigned to each factor. Thus, the predictive scoring system was constructed by summing the calculated values for each variable. A combined ROC analysis was further used to determine whether the scoring system had an elevated predictive accuracy for peritoneal metastasis in GC. As expected (Fig 3), compared to any other preoperative variables such as lymphatic invasion (0.726, 95% CI 0.677–0.774), depth of invasion (0.644, 95% CI 0.597–0.692), pathological type (0.539, 95% CI 0.483–0.595), and PLR (0.599, 95% CI 0.543–0.656), our score system, which had a higher AUC value (0.769, 95% CI 0.728–0.809), showed a more reliable discrimination ability as a predictive indicator for peritoneal metastasis in GC.

## Discussion

Peritoneal metastasis is usually associated with a poor prognosis in patients with GC[3]. It may be ineffectual for patients with peritoneal metastasis to merely undergo gastrectomy and regional lymphadenectomy. However, conversion therapy[6] and intraperitoneal hyperthermochemotherapy,[17] which could make follow-up surgery possible, are widely perceived as effective replacement therapies because they can prolong survival. Therefore, an accurate preoperative diagnosis is significant to determine an individualized treatment strategy.

At present, imaging techniques such as ultrasonography and CT are the most commonly used means to predict peritoneal metastasis. However, they are not reliable in accurately predicting peritoneal metastasis because peritoneal metastases are not always detectable. The sensitivity of ultrasonography is only 0.09 (95% CI, 0.03–0.21), whereas that of CT is 0.33 (95% CI, 0.16–0.56)[7]. By contrast, PET-CT has a higher predictive value in diagnosing peritoneal metastasis[18]. Its high cost has unfortunately greatly limited its prevalence. Current developments in laparoscopy have made it possible to examine the whole abdominal cavity[19]. However, it is expensive and unnecessary to perform laparoscopic examinations for all patients with GC because there are no clear signs to screen for peritoneal metastasis. Therefore, because of the integrated technical complexity and cost, there remains an urgent need to develop a more effective method to preoperatively diagnose peritoneal metastasis in patients with GC.

Blood tumor markers such as CEA and CA-199 are commonly used clinically today to help diagnose gastrointestinal tumors. However, the sensitivity of these indicators for diagnosing peritoneal metastasis is insufficient (CEA, 23.91%; CA-199, 36.96%)[10]. Thus, it remains controversial whether they can be used as reliable markers to predict peritoneal metastasis. On the other hand, Hwang et al. demonstrated that CA-199 had a preoperative predictable value for diagnosing peritoneal metastasis in patients with GC[20]. However, their findings are contrary to the results of our study, which indicated that the preoperative levels of CA-199 and CEA were completely unrelated to peritoneal metastasis.

Therefore, the relationship between a tumor and the body inflammatory status has received increasing attention[11,21,22]. The lymphocyte infiltration induced by the chronic inflammatory response of the tumor and its surrounding tissues contributes to tumorigenesis[23]. Research has also confirmed that the invasion of malignant tumor cells is related to the specific characteristics of tumor cells and depends on the microenvironment[24], especially the interaction of various inflammatory factors[25], which can also have an important influence on peritoneal metastasis. The mechanism needs further study. It is most likely that inflammatory mediators and cytokines secreted by inflammatory cells can stimulate the body to produce a series of stress responses, thereby resulting in excessive aggregation of inflammatory cells, oxidative damage, and other negative biological effects. This action ultimately promotes the transformation from normal cells to tumor cells and enhances the invasion and metastasis of a tumor.

Based on the aforementioned information, we focused on the significance of systemic inflammatory response markers that would be convenient and inexpensive to detect preoperatively. Neutrophils can suppress the activity of immunologic effector cells such as natural killer cells and lymphocytes to inhibit the immune system[26,27]. However, neutrophils could promote the formation and development of tumors by producing vascular endothelial growth factor and matrix metalloproteinase-9 [28,29]. Several studies have found that platelets are activated in patients with GC and that the level of activated platelets was intimately related to the severity of GC[24,30]. Growth factors, which are secreted by activated platelets, contribute to tumor progression. The activated platelets could promote the growth and immune escape of tumor cells, and thus enhance the proliferation and motility of tumors[31]. Monocytes are associated with prognosis in various types of tumors. The preoperative level of monocytes could be closely associated with the survival of T3N0M0 rectal cancer without neoadjuvant chemoradiotherapy[32]. Furthermore, the preoperative circulating monocyte level has also been identified as an independent risk factor for breast cancer-related death. Research has also reported that tumor-infiltrating T cells have the potential to stimulate monocyte to produce matrix metalloproteinase-2, matrix metalloproteinase-9, and vascular endothelial growth factor, which have significant roles in angiogenesis, invasion, and metastasis[33]. In contrast to the aforementioned systemic inflammatory response markers, lymphocytes are a major antitumor factor[34]. Immunoreactive cells, which are an important component of the tumor-specific immune response, participate in the specific killing effect on the tumor. An investigation[35] showed that lymphocytes could reflect the body’s tumor-resistant ability and could reduce metastasis and recurrence in the early stage of a tumor. Based on the aforementioned information, neutrophilia, thrombocytosis, mononucleosis, and lymphopenia could potentially be used to evaluate the malignant degree and predict a poor prognosis. Moreover, several other systemic inflammatory response markers have been studied in various types of cancers. Among these markers, the NLR, PLR, and MLR—each of which can be easily calculated—have shown great potential. Several research studies[36,37] showed that PLR and NLR can help in the diagnosis of malignant tumors and in predicting prognosis. However, few studies have used the PLR and MLR as predictors of peritoneal metastasis.

In the present study, we first tried to determine whether preoperative circulating monocytes, the PLR, and the MLR could be used to predict peritoneal metastasis. The neutrophil, platelet, and monocyte counts were higher, whereas the lymphocyte count was lower, in the peritoneal metastasis group than in the non-peritoneal metastasis group; however, the differences in the neutrophil and monocyte counts were not statistically significance. For this reason, we speculated that peritoneal metastasis may not be sufficiently sensitive to reflect these differences and the number patients with peritoneal metastasis who were enrolled in the study was insufficient. The PLR and NLR, which amplify single distribution differences, were fortunately significantly different between the two groups, as was expected. The ROC curve further indicated that the PLR and NLR were better predictive markers for peritoneal metastasis with a higher sensitivity and specificity.

No previous study has evaluated the relationship between the PLR and peritoneal metastasis; therefore, we determined the cutoff value by using ROC curves. Thus, patients were divided into the high PLR/NLR group and the low PLR/NLR group. Further study found that the NLR and PLR were closely associated with age and several tumor characteristics. This finding was consistent with a previous study showing that older age was an independent risk factor for high NLR[38]. However, one study[39] also demonstrated that peritoneal metastasis occurred more easily in young patients with GC than in elderly patients with GC (17.4% vs. 6.5%). The specific mechanism by which a high a NLR/PLR is associated with peritoneal metastasis is unclear. This may be because of age-related dysfunction of body immune anti-inflammatory ability and immunosurveillance for cancers. Moreover, an elevated NLR/PLR was statistically associated with a larger tumor size, deeper tumor invasion, ulcerative type, and higher level of lymphatic invasion. This finding may be because the elevated NLR/PLR, which is a better indicator of inflammatory status, reflected the body’s weakened defense ability and the destruction of the barrier against malignant tumor cells. This factor ultimately leads to a poor prognosis such as peritoneal metastasis of these patients. NLR contributes to the preoperative diagnosis of peritoneal metastasis and preoperative staging.

To make a more accurate preoperative diagnosis for peritoneal metastasis, we also investigated the significance of tumor characteristics such as tumor size, pathological type, histopathological differentiation, which could be easily obtained by preoperative endoscopy. Our ROC analysis demonstrated that significant predictors such as tumor size and pathological type did have predictive value for peritoneal metastasis. However, similar to the aforementioned preoperative serum markers, the diagnostic accuracy of each single parameter was insufficient. Therefore, we performed a multivariable logistic regression analysis. Through this, we identified the PLR, invasion depth, lymphatic invasion, and pathological type as the preoperative peritoneal metastatic predictors.

A combined preoperative score system that included the aforementioned tumor characteristics and inflammatory index was ultimately constructed. A combined ROC analysis was later performed to demonstrate whether the scoring system had improved diagnostic accuracy for peritoneal metastasis. As expected, the scoring system, which consisted of independent preoperative predictors, improved the accuracy (AUC = 0.769) with higher sensitivity (84.09%) and specificity (82.63%). Thus, we concluded that the preoperative score system has a potential diagnostic value for peritoneal metastasis.

Before this study, several other predictive systems had been constructed to predict peritoneal metastasis. For example, a predictive equation, which included the variables of tumor size, tumor stage, lymph node invasion, and histological differentiation, had a specificity and sensitivity of 78.3% and 88.5%[40]. However, it is difficult to preoperatively detect the tumor stage and lymph node invasion. Moreover, cancer and peritoneal metastasis are a systemic disease; therefore, all variables involved were tumor characteristic parameters, which cannot reflect the general status of patients. Another study[10] combined lysyl oxidase, CEA, CA-724, CA-199, and CA-125 to predict peritoneal metastasis with a sensitivity of 91.30%. However, it is uncommon and expensive to detect lysyl oxidase. Furthermore, the credibility of that study was insufficient: it enrolled only 113 patients with GC (67 patients without peritoneal metastasis and 46 patients with peritoneal metastasis).

Compared to these studies, our score system, which combined convenient preoperative values such as the depth of invasion, pathological type, lymphatic invasion, and PLR values, provided a credible predictive power (AUC = 0.781, 95% CI 0.721–0.841).Therefore, taking clinical practicability and accuracy together, our system could serve as a credible tool to predict peritoneal metastasis preoperatively, especially in imaging-negative patients.

This present study still has some limitations that should not be neglected. This was a retrospective study; therefore, tumor characteristic parameters such as invasion depth, lymphatic invasion, and pathological type were obtained postoperatively. These parameters could be obtained preoperatively by endoscopy; however, the results may be inaccurate. Futhermore, because of the incomplete data and the partial loss of our follow-up data, we did not add the analysis of prognosis in our study. Moreover, only 101 patients with peritoneal metastasis were enrolled in our study and all of these patients were from a single hospital. Therefore, our findings still require validation by a large prospective multicenter studies. Most importantly, the sensitivity of our score system was not sufficiently high to diagnose peritoneal metastasis. Therefore, it can only be used as an auxiliary tool.

## Conclusion

This is the first study that has attempted to investigate the relationship between the PLR and peritoneal metastasis. We found that the PLR, which was independently associated with peritoneal metastasis, could be used to predict the condition of peritoneal metastasis and thereby guide individualized treatment. Furthermore, the PLR combined with tumor characteristics such as invasion depth, lymphatic invasion, and pathological type could obviously improve the diagnostic accuracy. Moreover, this new scoring system is an economical and convenient tool that could be used to predict peritoneal metastasis, which will have an important role in imaging-negative patients.

## Supporting information

S1 File Minimal data. (XLSX)
